# NTF2-like domain of Tap plays a critical role in cargo mRNA recognition and export

**DOI:** 10.1093/nar/gkv039

**Published:** 2015-01-27

**Authors:** Jun Katahira, Lyudmila Dimitrova, Yumiko Imai, Ed Hurt

**Affiliations:** 1 Biomolecular Networks Laboratories, Graduate School of Frontier Biosciences, Osaka University, 1-3 Yamadaoka, Suita, Osaka 565-0871, Japan; 2 Department of Biochemistry, Graduate School of Medicine, Osaka University, 2-2 Yamadaoka, Suita, Osaka 565-0871, Japan; 3 Biochemie-Zentrum der Universität Heidelberg, Im Neuenheimer Feld 328, Heidelberg D-69120, Germany; 4 Department of Biological Informatics and Experimental Therapeutics, Graduate School of Medicine, Akita University, Akita 010-8543, Japan

## Abstract

Metazoan Tap-p15 (also called Nxf1-Nxt1) and yeast Mex67-Mtr2 heterodimers are the general mRNA export receptors. The RNA binding activity of Tap-p15, which is essential for mRNA nuclear export, has been attributed to the amino-terminal RNA binding module of Tap consists of RNA recognition motif (RRM) and leucine-rich repeat. In this study, we identified a novel RNA interaction surface in the NTF2-like (NTF2L) domain of Tap, which is analogous to the rRNA binding platform of Mex67-Mtr2. Tap-p15 uses the three domains to tightly bind the retroviral constitutive transport element. The RNA binding through the NTF2L domain is functionally relevant as introduction of mutations in this region reduced CTE-containing mRNA export activity. In contrast, only when the RRM and NTF2L domains were mutated simultaneously, bulk poly (A)^+^ RNA export and *in vivo* poly (A)^+^ RNA binding activities of Tap-p15 were significantly attenuated. Moreover, an engineered human cell line harboring the NTF2L domain mutation in the *NXF1* gene showed a synthetic growth phenotype and severe mRNA export defect under Aly/REF and Thoc5 depleted condition. These data suggest that Tap-p15 recognizes bulk mRNAs through combinatorial use of the distinct RNA binding domains.

## INTRODUCTION

Soluble macromolecules, such as RNAs and proteins, are transported through the nuclear pore complexes (NPCs), huge protein assemblies that span the nuclear envelope. Transport receptors specifically recognize the nuclear import and export signals of cognate cargoes and facilitate their translocation through NPCs using interactions with phenylalanine-glycine (FG-) repeats of nucleoporins, which form a meshwork inside NPCs (1,2). Importin/karyopherin-β-type proteins constitute a major nucleo-cytoplasmic transport receptor family, which carry a variety of cargoes into or out of the nucleus. The family members are subdivided into importin- and exportin-clades based on the directionality of the transportation process they mediate. A small GTPase Ran-GTP dictates the direction of the transport by regulating the assembly (for exportins) and disassembly (for importins) of the cargo-transport receptor complexes (3–7).

The importin/karyopherin-β family members Exportin-5 and exportin-t transport microRNAs and tRNAs from the nucleus to the cytoplasm, respectively (5). Structural studies of the cargo-transport receptor complexes have revealed the detailed molecular mechanisms of the cargo recognition, which include specific interactions of the RNA molecules and the corresponding transport receptors (8,9). Exportin-5, for example, along with bound Ran-GTP, forms a U-shaped conformation and recognizes the common structure of pre-miRNAs, i.e. a hairpin-like double-stranded structure with 2-nt overhang at the 3′-end. The double-stranded region of pre-miRNA is grabbed by Exportin-5 through the electrostatic interactions between the negatively charged backbone phosphates of pre-miRNA and the positively charged inner surface of the Exportin-5 molecule. In addition, a tunnel-like structure at the bottom of the U-shaped Exportin-5 accommodates the 2-nt overhang at the 3′-end of pre-miRNA (9,10).

**Figure 1.**
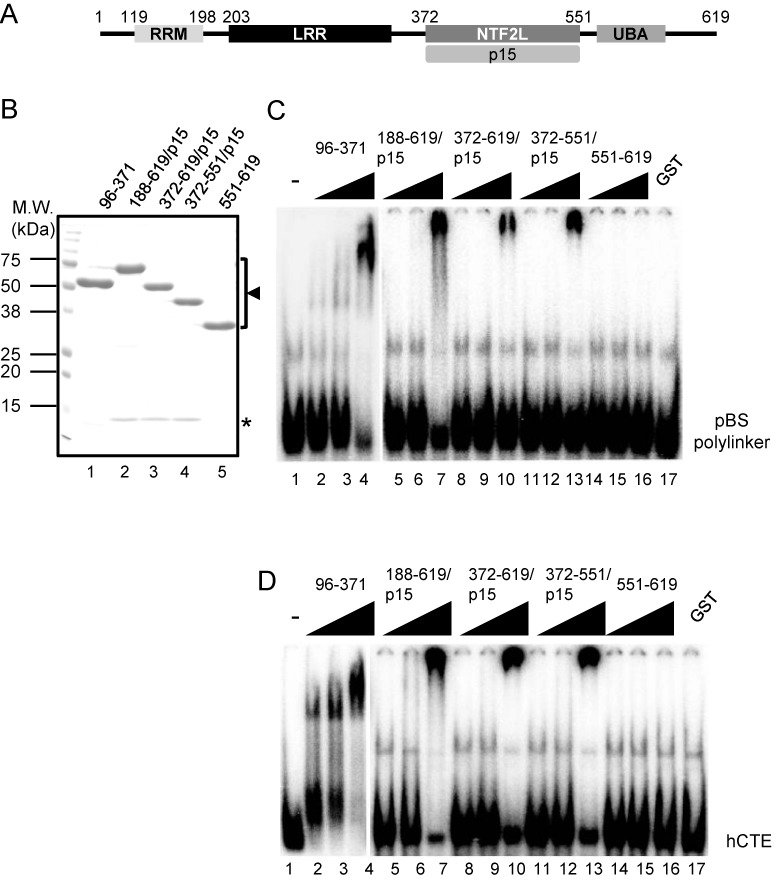
(**A**) Domain organization of Tap. Boxes indicate RNA recognition motif (RRM), leucine-rich repeat (LRR), NTF2-like (NTF2L) domain and ubiquitin-associated (UBA) domain. Numbers above the schema represent amino-acid positions of Tap. The NTF2L domain of Tap binds p15. (**B**) Purified recombinant proteins were analyzed by SDS-PAGE followed by CBB staining. Note that recombinant proteins GST-Tap (188–619), GST-Tap (372–619) and GST-Tap (372–551), which contain the NTF2L domain, were expressed and purified as complexes with p15. GST-Tap (96–371) and GST-Tap (551–619) lacking the NTF2L domain were expressed and purified as monomeric forms. The positions of Tap fragments and p15 are indicated by an arrowhead and an asterisk, respectively. Positions of molecular weight markers are shown on the left in kDa. (**C, D**) RNA-binding assay was performed using [^32^P]-labeled RNAs encoding the pBluescript SK polylinker sequence (C) or the halfmer sequence of the retroviral CTE (31) (D). Increasing amounts of the purified proteins shown in (B) (2, 6, 18 pmol; lanes 2–16) were added to total 10 μl of each binding reaction. GST was used as negative control (18 pmol; lane 17). Probe alone was run in lane 1. The binding reactions were separated by electrophoresis through 5% polyacrylamide gels and visualized by autoradiography. Relevant areas of each gel are shown as composites.

Nuclear export of mRNA is unique in that importin/karyopherin-β family proteins are not directly involved, and instead, the metazoan Tap-p15 and yeast Mex67-Mtr2 heterodimers function as general export receptors (5,11–13). As inferred from the overall structural similarity, Tap-p15, at least partially, rescues the lethal *mex67*/*mtr2* double knockout, indicating that the mechanism of mRNA export is evolutionarily conserved (14,15). Tap and Mex67 share a modular domain organization that includes the amino-terminal RNA recognition motif (RRM) followed by leucine-rich repeat (LRR), NTF2-like (NTF2L) middle domain that binds to p15 or Mtr2 and the ubiquitin associated (UBA) domain (Figure 1A) (16). Both the NTF2L and UBA domains contain an FG-repeat binding site, and are, thus, required for NPC translocation (17–19). As detected by both *in vivo* and *in vitro* assays, Tap-p15 and Mex67-Mtr2 also bind to RNA. The RRM domain of Tap exhibits non-specific RNA binding activity (20). However, the corresponding region of Mex67 is less well conserved than the other domains (21,22), and, in fact, it was unable to bind to RNA (20). A yeast-specific extra loop in the NTF2L scaffold of Mex67, which is located on the opposite side of the FG-repeat binding site, is the only known RNA binding region (15). Therefore, a fundamental question as to how Tap-p15 and Mex67-Mtr2 recognize cargo mRNA has yet to be fully elucidated.

As a compensation mechanism for the mostly non-specific nature of the intrinsic RNA binding activities, Tap-p15 and Mex67-Mtr2 interact with a series of adaptor proteins, which are recruited to mRNAs during transcription and processing (5,12,13,23,24). The RNA binding component of the transcription-export (TREX) complex, such as metazoan Aly/REF, and a couple of serine/arginine-rich (SR) proteins bind to the RRM domain of Tap through their arginine- and glycine-rich region (25–28). In addition, Thoc5, another RNA binding component of the metazoan TREX complex, interacts with the NTF2L domain of Tap (29). Through these protein–protein interactions, Tap-p15 selects mRNAs as cargoes, which are structurally far more divergent than the other small non-coding RNAs.

In contrast to cellular mRNAs, certain viruses that replicate in the nucleus directly exploit Tap-p15. The *cis*-acting RNA sequence called constitutive transport element (CTE) of the D-type retrovirus, which forms a 2-fold symmetrical stem-loop structure, recruits Tap-p15 to facilitate nuclear export of unspliced viral mRNA (30). The RRM and LRR domains of Tap show structurally and biochemically similar properties to the spliceosomal U2B’’ and U1A’ heterodimer and act as a specific CTE binding module (20). A recent structural study revealed the molecular mechanism of the interaction of one symmetrical unit of CTE (halfmer CTE; hereafter referred as hCTE) and the CTE binding module. Indeed, both the RRM and LRR domains extensively interact with hCTE in the complex (31). However, there still remains a possibility that the carboxyl-terminal region of Tap and/or p15 may also participate in CTE recognition, since the CTE binding module interacts with only one face of hCTE, leaving the other face exposed (16).

In this study, we show that Tap has an additional RNA binding site in the NTF2L domain. The novel RNA binding site became apparent upon heterodimerization with p15. A fragment of Tap consisting of the RRM, LRR and NTF2L domains complexed with p15 bound to hCTE with higher affinity than the individual RNA binding domains, indicating that these domains function cooperatively to recognize CTE-containing RNA. In fact, Tap derivatives containing mutations in the RNA binding surface of the NTF2L domain exported a CTE-containing reporter mRNA less efficiently than the wild-type protein. Contrary to these observations, simultaneous mutations in the NTF2L and RRM domains, but not single mutations in each domain, affected the cellular poly (A)^+^ RNA binding and export activities of Tap-p15. Notably, a human cell line harboring the *nxf1* gene with the NTF2L domain mutation exhibited a synthetic growth phenotype and severe mRNA export defect on Aly, and to a lesser extent, on Thoc5 depletion. Our data suggest that cellular mRNAs are allocated to the distinct RNA binding domains of Tap-p15 by the different adaptor proteins and that bulk mRNAs are recognized by combinatorial use of the distinct RNA binding domains.

## MATERIALS AND METHODS

### Plasmids

A cDNA fragment encoding Tap (96–551) was amplified by polymerase chain reaction (PCR) and cloned to the BamHI-XhoI site of the pGEX6P3 vector. All the other *Escherichia coli* expression vectors have been reported previously (14,29). A cDNA fragment encoding the wild-type full-length Tap was amplified by PCR and cloned to the BglII-EcoRI site of the pEGFP-C1 vector (Clontech). The resulting vector was named pEGFP-Tap. Alanine scan mutations in the NTF2L domain (29) and R^128^K>EE mutations in the RRM (20) were introduced by the QuikChange kit (Stratagene). A siRNA-resistant wild-type Tap (Tap^R^) expression vector was constructed by introducing silent mutations to the siRNA target site (TCT ATC ATC ATC > agc ATt ATa ATt: lower case letters indicate mutations) of the pEGFP-Tap vector using the QuikChange kit. All the mutations were verified by DNA sequencing. A mammalian expression vector of p15-FLAG has been described previously (32). A CTE containing *Renilla* luciferase reporter and a firefly luciferase control plasmids have been described (33). A plasmid vector encoding the Simian retrovirus-D serotype 1 (SRV-1) CTE (pBS-CTE) is a gift from Dr S. Shibata. Genomic DNA fragments of human *NXF1* gene were obtained by PCR. The amplified genomic DNA fragments and a Tap cDNA fragment harboring the m8 mutation along with the bovine growth hormone polyadenylation signal (BGHpA) were cloned to the pNT1.1 vector (34) to obtain pNT1.1-m8. The pGK-Neo cassette in pNT1.1-m8 was replaced by the pGK-Puro cassette to obtain pNT1.1-Puro-m8 (Figure 6A). An annealed oligonucleotide pair encoding the guide RNA sequence (5′-caccgttcgtatcacacacttact-3′, 5′-aaacagtaagtgtgtgatacgaac-3′) was cloned to the pX330 vector (Addgene) to obtain pX330-Tap6.

**Figure 2.**
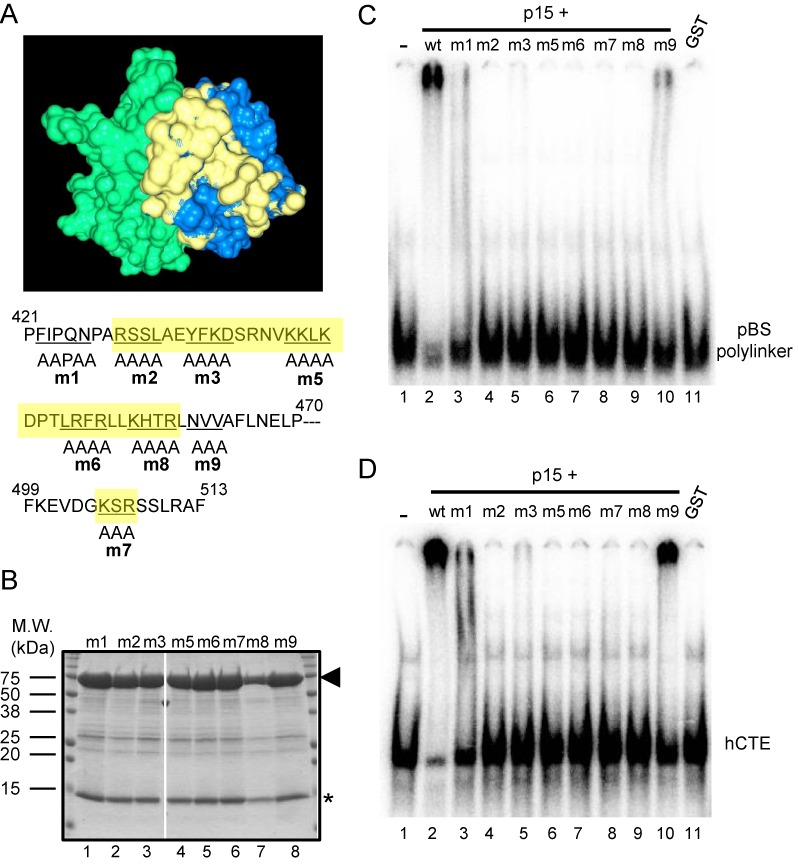
(**A**) Surface representation showing the NTF2-like domain of Tap (blue) complexed with p15 (green) (39). The regions of Tap critical for the RNA binding are colored in yellow. The positions of alanine-scan mutations are indicated at the bottom of the figure as a single-letter code. The numbers on top of the sequence indicate the amino-acid positions of Tap. The regions colored in yellow in the 3D model are shaded in yellow; see (13) for more details. (**B**) Purified recombinant GST-Tap (188–619) proteins harboring the alanine-scan mutations complexed with p15 were analyzed by SDS-PAGE followed by CBB staining. The positions of Tap (188–619) and p15 are indicated by an arrowhead and an asterisk, respectively. Positions of molecular weight markers are shown on the left in kDa. (**C, D**) RNA-binding assay was performed using [^32^P]-labeled RNAs encoding pBluescript SK polylinker sequence (C) or hCTE (D). Purified recombinant GST-Tap (188–619) or the mutants complexed with p15 (lanes 2–10) or GST alone (lane 11) (18 pmol each) were added to total 10 μl of each binding reaction. Probe alone was run in lane 1. The binding reactions were analyzed by electrophoresis through 5% polyacrylamide gels and visualized by autoradiography.

**Figure 3.**
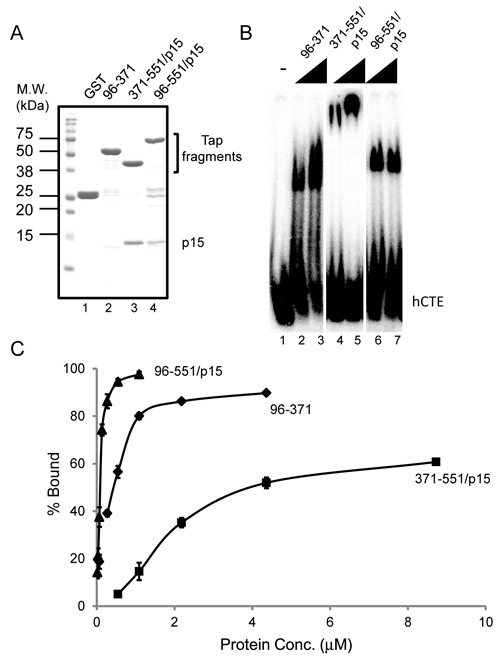
(**A**) Proteins subjected to CTE band shift assay. GST-Tap (96–371) was purified as a monomer, whereas GST-Tap (371–551) and GST-Tap (96–551) were purified as heterodimers with p15. The purified proteins were separated by SDS-PAGE and visualized by CBB staining. The positions of the Tap fragments and p15 are indicated on the right. Positions of molecular weight markers are shown on the left in kDa. (**B**) RNA binding assay was performed using [^32^P]-labeled hCTE RNA probe. Increasing amounts of the purified proteins [Tap (96–371): 3, 6 pmol; Tap(371–551)/p15: 12, 24 pmol; Tap (96–551)/p15: 0.75, 1.5 pmol] were added to total 10 μl of each reaction. Probe alone was run in lane 1. (**C**) Quantification of the RNA binding assays. The plots indicate mean ± SD of three independent measurements.

**Figure 4.**
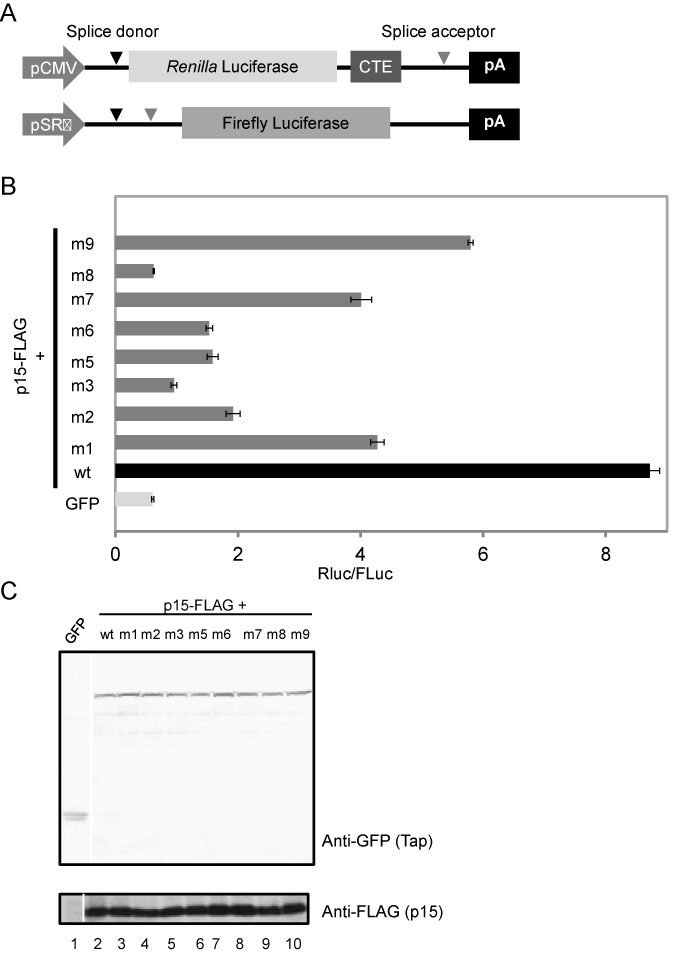
(**A**) Structures of pCMV128-RLucCTE (test plasmid) and pME-FLuc (control plasmid). (**B**) The test and the control plasmids along with pEGFP or pEGFP-Tap (full-length; wild type or the NTF2L domain mutants) were transfected to 293F cells. A p15-FLAG expression vector was included in the transfections as indicated. At 48 h post transfection, dual-luciferase assay was performed and RLuc/FLuc ratios were calculated. Note that upon overexpression of GFP-Tap and p15-FLAG, expression of RLuc was increased by ∼14-fold as compared with GFP (*P* = 1.11 × 10^−7^ by Student's *t*-test). The Tap mutants harboring the alanine-scan mutations in the NTF2L-domain (m1 to m9) activated RLuc expression less efficiently than the wild-type protein (*P*-values, m1: 2.55 × 10^−6^, m2: 4.90 × 10^−7^, m3: 1.94 × 10^−7^, m5: 3.06 × 10^−7^, m6: 2.12 × 10^−7^, m7: 4.09 × 10^−6^, m8: 1.09 × 10^−7^, m9: 7.13 × 10^−6^). (**C**) GFP or GFP-Tap (wild type or the NTF2L-domain mutants) expression plasmids used in (B) were transfected to 293 cells. A p15-FLAG expression vector was included in the transfections as indicated. At 48 h post transfection, total cell extracts were prepared and they were subjected to western blot using anti-GFP (upper panel) and anti-FLAG (lower panel) antibodies.

**Figure 5.**
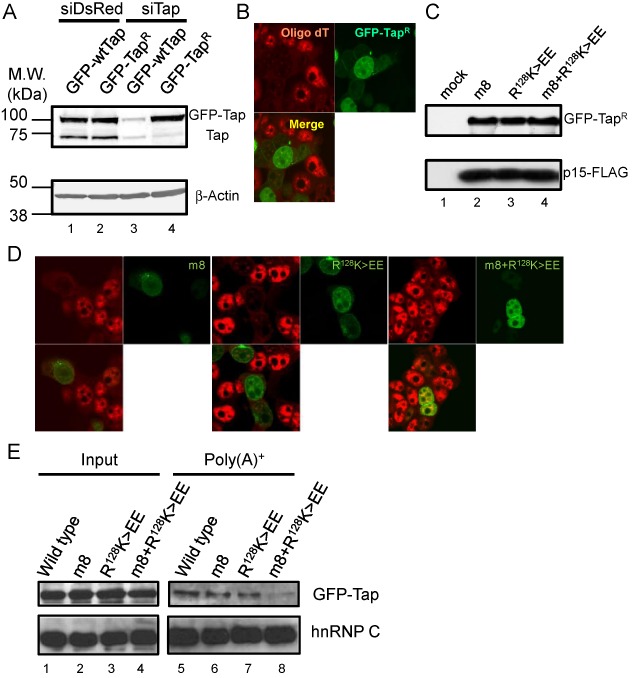
(**A**) Indicated siRNAs were transfected to 293F cells. At 36 h after the siRNA transfection, GFP-fusion vectors encoding wild type and siRNA-resistant Tap (Tap^R^) were transfected along with a p15-FLAG expression vector. At 24 h after the second transfection, total cell lysates were prepared and they were subjected to western blot using anti-Tap (upper panel) and anti-β-actin (lower panel) antibodies. Positions of molecular weight markers are shown on the left in kDa. (**B**) 293F cells treated as in (A) were fixed and subjected to *in situ* hybridization using Cy3-labeled oligo-dT_50_ probe. The cells were observed by a confocal microscopy. (**C**) GFP-Tap^R^ fusion vectors harboring point mutations in the NTF2L (m8) and RRM (R^128^K>EE) domains or both (m8+R^128^K>EE) were transfected to 293F cells along with a p15-FLAG expression vector. Total cell extracts prepared at 48 h post transfection were subjected to western blot using anti-GFP (upper panel) and anti-FLAG (lower panel) antibodies. (**D**) Same as in (B), but the GFP-Tap^R^ variants used in (C) were expressed instead of the wild-type protein. (**E**) 293F cells expressing the indicated GFP fusion proteins were irradiated with UV light. Whole cell extracts were prepared and poly (A)^+^ RNA was purified by oligo-dT cellulose chromatography. RNase A-treated whole cell extracts (input) and poly (A)^+^ RNA fractions were analyzed by western blot using anti-GFP (upper panels) and anti-hnRNP C (lower panels) antibodies.

**Figure 6.**
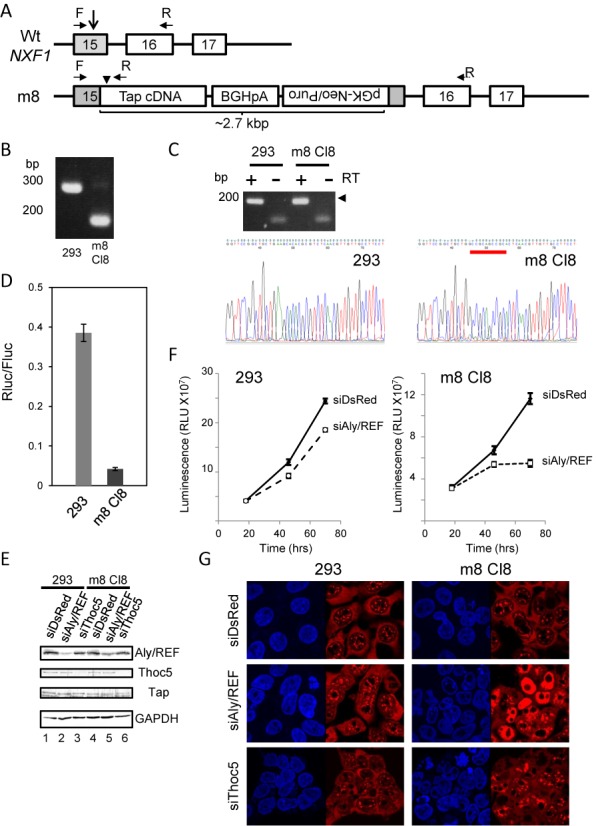
(**A**) CRISPR/Cas9-mediated genome editing of the *NXF1* locus. A fragment of Tap cDNA harboring m8 mutation was introduced in-frame to the 15th exon (indicated by a gray box) of the wild-type *NXF1* gene. Two donor vectors harboring either pGK-Neo or pGK-puro selection cassette were used simultaneously to facilitate editing of multiple alleles. Boxes with numbers and thin lines indicate the exons and introns, respectively. A vertical arrow indicates the position of CRISPR/Cas9-mediated cleavage site. BGHpA indicates the bovine growth hormone gene polyadenylation signal. A vertical arrow head indicates the position of m8 mutation. Horizontal arrows show the positions of the forward (F) and reverse (R) primers used in panels (**B**) and (**C**). (B) PCR using genome DNAs isolated from each cell line revealed successful homologous recombination event in the m8 cell line. (C) The successful gene editing was confirmed by direct sequencing of RT-PCR amplicons (arrow head), which shows the expected mutations in the m8 cell line (indicated by red line). (**D**) CTE export assay was performed using the wild-type 293F and m8 cell lines. (**E**) The wild-type and m8 cell lines were treated with the indicated siRNAs for 72 h. The expression of Aly/REF, Tap and GAPDH was detected by western blot. Relevant areas of each blot are shown as composites. (**F**) The wild-type and m8 cell lines were treated with the indicated siRNAs. Growth of the cells under each condition was examined by a luminescent-based cell viability assay. (**G**) The wild-type and m8 cell lines were treated with the indicated siRNAs. The cells were fixed and subjected to *in situ* hybridization using Cy3-labeled oligo-dT_50_ probe.

### Protein expression and purification

GST and GST-Tap fusion proteins were expressed in the *E. coli* BL21(DE3) codon plus strain and purified by glutathione-Sepharose gel chromatography (GE Healthcare) as described previously (29). Untagged p15 was co-expressed as indicated. GST-Tap (96–551) complexed with p15 was further purified by heparin-agarose gel chromatography (GE Healthcare).

### RNA probe preparation and gel shift assays

To obtain a template for the halfmer sequence of SRV-1 CTE, pBS-CTE was amplified by PCR using oligonucleotides 5′-GCTAATACGACTCACTATAGGGTCACTAACCTAAGACAGGAGGG-3′ and 5′-TCACTTTTACCCGTCTTTGGATTAGGC-3′ (underline indicates the T7 promotor sequence). A template for an RNA probe encoding the polylinker sequence was prepared by linearizing the pBluescript (pBS) vector by NotI. Run-off transcription was performed in the presence of [^32^P]-UTP using Riboprobe Combination system according to the manufacturer's protocol (Promega). The radio-labeled RNA probes were purified by gel electrophoresis. RNA gel shift assays were performed as previously described (29,35).

### Cell culture and transfection

Human 293F cells (Invitrogen) were cultured in Dulbecco's modified Eagle's medium supplemented with 10% fetal bovine serum. Transfection of plasmids and siRNAs was done as previously described (29).

### Luciferase reporter assays

Luciferase reporter assays were performed using the dual luciferase reporter assay system according to the manufacturer's protocol (Promega). Three independent transfections were done for each combination. Significance of difference was tested by Student's *t*-test.

### Cell growth assay

Cell growth was examined by luminescent-based cell viability assay using CellTiter-Glo assay kit according to the manufacturer's protocol (Promega).

### CRISPR/Cas9-mediated genome editing

The Cas9 and guide RNA expression vector pX330-Tap6 and the two linearized donor vectors pNT1.1-m8 and pNT1.1-Puro-m8 were simultaneously transfected to 293 cells. Selection for G418- and puromycin-resistance was started at 48 h after transfection. Genomic DNA was obtained from individual colonies and successful homologous recombination was confirmed by PCR. Total RNA obtained from the wild-type and m8 clone 8 cells was subjected to RT-PCR. Successful introduction of m8 mutation was further confirmed by directly sequencing the RT-PCR product.

### siRNAs

The sequences of siRNAs against dsRed, Thoc5, Aly/REF and Tap have been described previously (29).

### UV-crosslinking

UV-crosslinking experiments were performed as previously described (14,32). Oligo-dT cellulose was purchased from WAKO chemicals.

### Oligo-dT *in situ* hybridization

*In situ* hybridization using Cy3-labeled oligo-dT_50_ probe was performed as previously described (29).

### Antibodies

Antibodies against Tap and Thoc5 have been described (14,29). Anti-GFP (Invitrogen), -Aly/REF (Abcam), -GAPDH (Ambion), -FLAG peptide, -hnRNP C and -β-actin (Sigma) antibodies were commercially acquired.

## RESULTS

### NTF2L domain of Tap shows an RNA binding activity

Tap exhibits a modular domain organization that includes the RRM, LRR, NTF2L and UBA domains (Figure 1A). As has been reported previously (20), purified recombinant CTE binding module of Tap [Tap (96–371)] (Figure 1B, lane 1) that consists of the RRM and LRR domains bound to RNA probes encoding the pBS polylinker or the hCTE sequences in RNA gel shift assays (Figure 1C and D, lanes 2–4). In contrast, Tap (188–619) lacking the RRM domain did not bind to the RNAs (data not shown; see (14)). However, we, by chance, noticed that Tap (188–619), if applied as a complex with p15 (Figure 1B, lane 2; co-purified p15 is indicated by an asterisk), bound to the RNA probes (Figure 1C and D, lanes 5–7). We thus revisited the RNA binding activity of Tap-p15 in more detail. For this purpose, recombinant Tap (372–619)-p15, Tap (372–551)-p15 and Tap (551–619) were prepared (Figure 1B, lanes 3–5) and subjected to RNA gel shift assays. By comparing the RNA band shift patterns, we concluded that the RNA binding activity was attributable to the NTF2L domain complexed with p15 (Figures 1C and 1D, lanes 11–13). Previously, we have shown that a surface of the NTF2L domain of Tap, which is in close proximity to the RNA binding loop of the yeast homolog Mex67, binds to Thoc5, an mRNA binding adaptor protein (29) (Figure 2A). To examine whether the same surface is responsible for the RNA binding activity in our assays, a series of Tap (188–619) derivatives harboring alanine substitutions in the NTF2L domain were prepared as complexes with p15 (Figure 2B; see also Figure 2A for the positions of the mutations). As shown in Figure 2C and D, the mutants bound to the RNAs less efficiently. Thus, our data indicate that the surface of the NTF2L domain of Tap, which is stabilized in the complex with p15, is a novel RNA binding site.

### Different RNA binding domains of Tap function cooperatively in CTE-mediated export

The above data indicate that Tap-p15 contains three independent RNA binding domains; i.e. the RRM, LRR and NTF2L domains. To examine the functional relationship between them, we prepared various recombinant proteins harboring different combinations of the RNA binding domains (Figure 3A) and compared their hCTE binding activities. As shown in Figure 3B, Tap (96–371) and Tap (371–551)-p15 gave different band shift patterns (compare Figure 3B, lanes 2 and 3 with lanes 4 and 5). Furthermore, the fragment containing all of the RNA binding domains complexed with p15 [Tap (96–551)-p15] showed a distinct band shift pattern; the RNA–protein complex migrated at the position in between the two other complexes (Figure 3B, lanes 6 and 7). The observation suggests that Tap (96–551)-p15 interacts with hCTE in a different manner. Quantification of the RNA binding efficiency revealed that Tap (96–551)-p15 showed the highest affinity to hCTE (Figure 3C). These data indicate that the distinct RNA binding domains of Tap-p15 function cooperatively. We found that *Chaetomium thermophilum* (Ct) Mex67-Mtr2 (29) (Supplementary Figure S1A and B) also bound to RNA through the separate domains (Supplementary Figure S1C). In this case, however, the NTF2L domain showed slightly higher affinity than the amino-terminal fragment containing the RRM and LRR domains (Supplementary Figure S1D).

### RNA binding through NTF2L domain is crucial for CTE-dependent RNA export

As previously reported, Tap-p15 facilitates CTE-driven mRNA export (30). To examine whether the RNA binding through the NTF2L domain is relevant for CTE-containing mRNA export, we performed dual luciferase assays using a CTE-containing reporter (31,33) (Figure 4A). As has been previously reported (31), overexpression of the wild-type GFP-Tap fusion protein along with p15 facilitated the nuclear export of CTE-containing mRNA, resulting in the activation of *Renilla* luciferase expression (Figure 4B, compare GFP with wild-type Tap-p15). In contrast, GFP-Tap derivatives containing point mutations in the NTF2L domain activated the CTE nuclear export less efficiently (Figure 4B). As evaluated by western blot, the levels of both the wild-type and the mutant GFP-Tap fusion proteins and p15 were similar (Figure 4C). Although the extent to which each mutation impeded the CTE export activity varied, overall the data correlated well to the *in vitro* CTE binding activities (Figure 2D), indicating that the RNA binding through the NTF2L domain is required for the CTE-dependent mRNA export.

### Redundancy of the RNA binding domains in bulk mRNA export

The importance of the three RNA binding domains for bulk poly (A)^+^ RNA export was also examined. To accomplish this, we set up a rescue experiment. As previously reported, when the expression of the endogenous Tap was knocked down, export of poly (A)^+^ RNAs was blocked (29). As expected, a GFP-Tap containing silent mutations in the siRNA target sequence (GFP-Tap^R^) was resistant to the siRNA treatment (Figure 5A). Overexpression of GFP-Tap^R^ along with p15 rescued the nuclear export block of poly (A)^+^ RNAs (Figure 5B). GFP-Tap^R^ variants with single mutations in either the RRM or NTF2L domains (i.e. R^128^K>EE and m8) and with double mutations in both sites (i.e. m8+ R^128^K>EE) were expressed in similar levels (Figure 5C). Although the single mutants R^128^K>EE and m8 rescued the poly (A)^+^ RNA export block, the double mutant m8+ R^128^K>EE did not do so efficiently (Figure 5D). Previously it was shown that the interaction of Tap-p15 with bulk poly (A)^+^ RNA *in vivo* can be detected by UV-crosslinking (14,32). Therefore, we performed poly (A)^+^ RNA pull-downs. The mutants m8 and R^128^K>EE were recovered in the poly (A)^+^ RNA fraction as efficiently as the wild-type protein (Figure 5E, lanes 6 and 7). In contrast, recovery of the double mutant in the poly (A)^+^ RNA fraction was severely impaired (Figure 5E, lane 8).

To further examine the importance of the NTF2L domain of Tap for bulk poly (A)^+^ RNA export, we established a 293F cell line expressing the NTF2L domain mutant. A fragment of the Tap cDNA containing the m8 mutation was introduced into the *NXF1* allele by CRISPR/Cas9-mediated genome engineering (36) (Figure 6A). After G418 and puromycin selection, a successful homologous recombination was confirmed by PCR analysis of genomic DNA (Figure 6B). We further confirmed that the m8 cell line exclusively expressed the mutant version of Tap by direct sequencing of the RT-PCR product (Figure 6C). As expected, the m8 cell line supported poorly the CTE-containing reporter gene expression (Figure 6D). Although the m8 cell line grew a little bit slower than the wild-type cells (Figure 6F), it did not show apparent poly (A)^+^ RNA export defect (data not shown; see Figure 6G). This is consistent with the results of the rescue experiments shown in Figure 5. We thought that the mRNA binding adaptor proteins might complement the attenuated poly (A)^+^ RNA binding ability of the NTF2L domain mutant. Indeed, the m8 cell line showed a synthetic growth phenotype due to severe poly (A)^+^ RNA export defect under Aly/REF-depleted condition (Figure 6E, F, G). In addition, when Thoc5 was depleted, poly (A)^+^ RNA export block was also observed, although in this case, the nuclear accumulation of poly (A)^+^ RNA was less pronounced than Aly/REF depletion (Figure 6G). In contrast, as reported previously by us and others (29,37,38), Aly/REF and Thoc5 depletion in wild-type cells did not severely affect their growth and poly (A)^+^ RNA export (Figure 6E, F, G). From these data, we concluded that the distinct RNA binding domains of Tap-p15 function redundantly in nuclear export of bulk cellular mRNAs.

## DISCUSSION

Human Tap-p15 and yeast Mex67-Mtr2 are the evolutionarily conserved general export receptors for most mRNAs (5,12,13,24). Tap-p15 and Mex67-Mtr2 can be crosslinked to bulk poly (A)^+^ RNAs *in vivo* (14,21,32). This suggests that the export receptors directly interact with cargo mRNAs *en route* to the cytoplasm. However, since the intrinsic RNA binding ability of the export receptors is mostly non-specific, a series of adaptor proteins, such as SR proteins and the TREX components Aly/REF and Thoc5, are thought to assist in selecting mRNA from the other RNA species as specific cargo (5,12,13,16,24). Due to such complexity, the mechanism by which these transport receptors recognize cargo mRNAs, which is a key to understand the mRNA export process, has not yet been fully elucidated. In this study, we identified an additional RNA binding site in the NTF2L domain of Tap, which becomes predominantly relevant upon heterodimerization with p15. Although we do not address the issue in the present study, our data do not exclude a possibility that in addition to the well-established function in maintaining the structural integrity of the NTF2L fold (39), p15 may also directly interact with RNA as proposed for the yeast counterpart Mtr2 (15). Since the RNA binding activity of CtMex67-Mtr2 is also attributed to the separate domains, the mechanism of RNA recognition by the mRNA export receptors appears to be evolutionarily conserved. In addition, our data indicate that the importance of the newly identified RNA binding domain of Tap-p15 is different for bulk cellular and viral CTE-containing mRNAs, respectively.

For the export of cellular mRNAs, Tap-p15 is thought to initiate recognition of cargo mRNAs via protein–protein interactions with the adaptor proteins (5,12,13,16,24). The binding to cargo mRNAs through the adaptor proteins compensates for the weak and non-specific RNA binding activity of Tap-p15 (40–43). The spatial proximity between the binding sites of the adaptor proteins to Tap-p15 and RNA may allow transfer of cargo mRNA to Tap-p15 (43). We have previously reported that Thoc5, a metazoan-specific RNA binding component of the TREX complex, binds to the NTF2L domain of Tap (29), which completely overlaps with the newly identified RNA binding site in this study. This suggests that Thoc5 also assists in recruiting mRNAs to the NTF2L domain. As has been revealed in various species, however, depletion of either Aly/REF or Thoc5 alone does not completely block the nuclear export of bulk poly (A)^+^ RNAs in metazoans (29,37,38,44). In line with the previous data, we show that point mutations in either RRM or the NTF2L domain of Tap do not completely inhibit the poly (A)^+^ RNA binding and export activities. In contrast, Tap harboring the double mutations can neither efficiently support bulk poly (A)^+^ RNA export nor bind to poly (A)^+^ RNAs *in vivo*. In addition, a 293F cell line expressing the NTF2L domain mutant of Tap showed a synthetic growth phenotype and poly (A)^+^ RNA export defect on Aly/REF and Thoc5 depletion. Moreover, double knock down of Thoc5 and Aly/REF in wild-type 293F cells induced nuclear accumulation of poly (A)^+^ RNA as previously reported (45) (Supplementary Figure S2). These data indicate that for the export of bulk cellular mRNAs, the separate RNA binding domains play a redundant role. It is conceivable that the recognition of cargo mRNAs through the use of the independent RNA binding domains and the different adaptors ensures the specificity and affinity to mRNA cargoes.

Retroviral CTE is thought to directly recruit Tap-p15. CTE contains internal loops arranged in 2-fold symmetry. Each symmetrical unit with a conserved AAGACA sequence in the internal loop accommodates one molecule of the Tap-p15 heterodimer (46,47). The structural study of the amino-terminal CTE binding module of Tap with one symmetrical unit of CTE (i.e. hCTE) revealed that both the RRM and LRR domains contact the sugar-phosphate backbone within and around the internal loop. The binding of RRM-LRR induces flip out of three residues in the internal loop of hCTE, allowing it to adopt an L-shaped conformation (31). Importantly, one face of hCTE in the complex still remains exposed in solution. Therefore, as pointed out previously (16), the NTF2L domain may interact with the exposed surface of hCTE and more compactly package it to facilitate the nuclear export. Previously, it has been reported that the nuclear export of CTE-containing mRNA requires the two FG-repeat binding sites of Tap, which reside in the NTF2L and UBA domains. But the two domains were not completely equivalent and the function of the NTF2L domain was only partially replaced by that of the UBA domain and vice versa. Thus, the CTE export activity of a Tap derivative harboring 2xUBA domains was reduced by ∼50% in comparison with the wild-type protein (48). The reduction in the CTE export activity is in accordance with our data that the NTF2L domain additionally has the RNA binding activity. In contrast to their data, however, our m8 mutant almost completely lost the CTE export activity. This may indicate that in the wild-type context, the binding of CTE through the NTF2L domain affects the overall structural integrity and the FG-repeat binding activity of the Tap-p15 heterodimer. Our recent structural analysis revealed that Tap (96–555)-p15 is able to form a 2-fold symmetrical platform via intimate domain-swapped dimerization. The structural model predicts that the RRM, LRR and NTF2L domains of Tap arranged in one face of the dimer fit in well with the symmetrical structure of the full-length CTE (see the accompanying paper by S. Aibara *et al.* for details). Intriguingly, the formation of the platform also allows alignment of the FG-repeat binding sites on the other side of the complex. Moreover, introduction of mutations to the residues critical for the dimer formation reduced the CTE binding activity of Tap-p15, suggesting a connection between the CTE binding and formation of the dimer. Therefore, the specialized dimeric platform configuration formed on CTE may promote the FG-repeat binding and accelerate the NPC translocation of CTE containing mRNA. The CTE export activities of the NTF2L domain mutants varied in the range from nearly 0 (m3 and m8) and 50% (m7) to 70% (m1 and m9) of the wild-type Tap-p15 activity (Figure 4B). These mutants bind to CTE through the RRM-LRR domains probably with a support of the attenuated RNA binding activity in the mutated NTF2L domain. Thus, we might simply observe the differences in the residual RNA binding activities, which could not be detected in our *in vitro* assay conditions. Although speculative, another plausible explanation for the observation could be that the mutations in the different sites of the NTF2L domain might have distinct effects on the formation of the dimeric platform that occurs upon binding to CTE and thus affect the CTE export activities in variable extent.

## SUPPLEMENTARY DATA

Supplementary Data are available at NAR Online.

SUPPLEMENTARY DATA
