# Supplementary material for: NTF2-like domain of Tap plays a critical role in cargo mRNA recognition and export
Source: Nucleic Acids Res. 2015 Jan 27;43(3):1894–904. doi: 10.1093/nar/gkv039 (PMC4330393; doi:10.1093/nar/gkv039)
Supplement: SUPPLEMENTARY DATA [file supp_43_3_1894__index.html]

NTF2-like domain of Tap plays a critical role in cargo mRNA recognition and export — SUPPLEMENTARY DATA 

# NTF2-like domain of Tap plays a critical role in cargo mRNA recognition and export

## SUPPLEMENTARY DATA

**Files in this Data Supplement:**

- Supplementary Figures Captions
- Supplementary Figures
