# Supplementary material for: NTF2-like domain of Tap plays a critical role in cargo mRNA recognition and export
Source: Nucleic Acids Res. 2015 Jan 27;43(3):1894–904. doi: 10.1093/nar/gkv039 (PMC4330393; doi:10.1093/nar/gkv039)
Supplement: SUPPLEMENTARY DATA [file supp_gkv039_nar-03380-r-2014-File003.docx]

**SUPPLEMENTARY DATA**

**Supplementary figure S1.** (A) Domain organization of *Chaetomium thermophilum* (Ct) Mex67. Boxes indicate RNA recognition motif (RRM), leucine-rich repeat (LRR), NTF2-like (NTF2L) domain and ubiquitin-associated (UBA) domain. Numbers above the schema represent amino acid positions of CtMex67. The NTF2L domain of CtMex67 binds to CtMtr2. (B) Purification of various domains of CtMex67. A CBB stained gel is shown. Positions of molecular weight markers are shown on the left in kDa. Positions of the CtMex67 fragments and CtMtr2 are shown on the right of the panel. (C) RNA binding assay was performed using [^32^P]-labeled halfmer CTE probes. Increasing amounts of the proteins (3, 6, 12, 24, 48 pmol) were added to total 10 μl of each reaction. Probe alone was run in lane 1. (C) Quantification of the RNA binding assays. The plots indicate mean + S.D. of three independent RNA binding reactions.

**Supplementary figure S2.** (A) 293F cells were treated with the indicated siRNAs for 60 hrs. Whole cell extracts were prepared from each culture and subjected to western blot using anti-Tap, anti-Thoc5, anti-Aly/REF and anti-GAPDH (lower panel) antibodies. (B) 293F cells treated with the indicated siRNAs for 60 hrs were fixed and subjected to *in situ* hybridization using Cy-3 labeled oligo-dT_50_ probe.
