# Supplementary material for: NTF2-like domain of Tap plays a critical role in cargo mRNA recognition and export
Source: Nucleic Acids Res. 2015 Jan 27;43(3):1894–904. doi: 10.1093/nar/gkv039 (PMC4330393; doi:10.1093/nar/gkv039)
Supplement: SUPPLEMENTARY DATA [file supp_gkv039_nar-03380-r-2014-File004.pptx]

## Slide 1
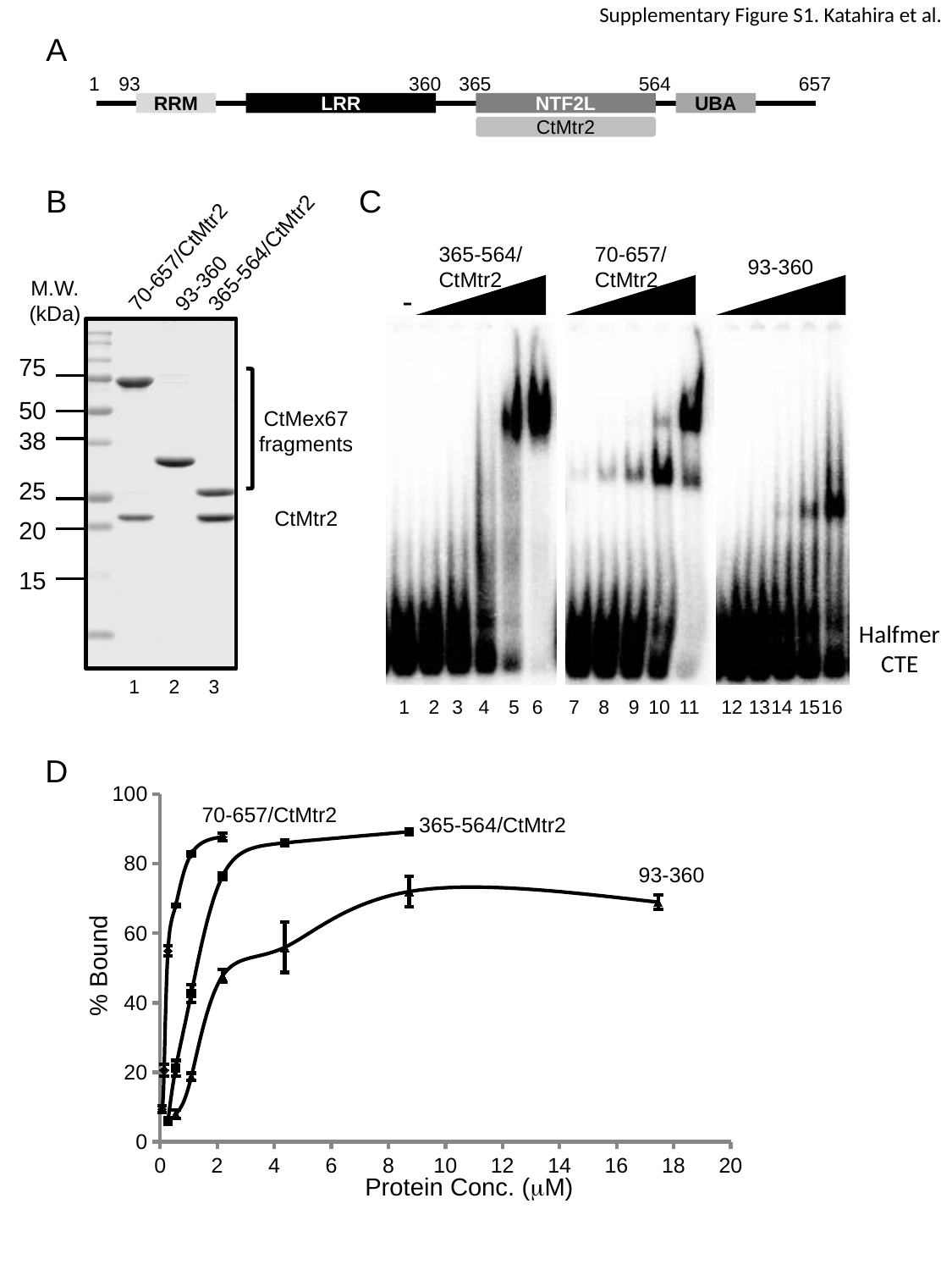

Supplementary Figure S1. Katahira et al.
A
1
93
360
365
564
657
RRM
LRR
NTF2L
UBA
CtMtr2
365-564/CtMtr2
70-657/CtMtr2
93-360
M.W.
(kDa)
75
50
CtMex67
fragments
38
25
CtMtr2
20
15
1
2
3
B
C
365-564/
CtMtr2
70-657/
CtMtr2
93-360
-
Halfmer
CTE
1
2
3
4
5
6
7
8
9
10
11
12
13
14
15
16
D
### Chart
| Category | deltaN | NTF2/Mtr | RBD |
|---|---|---|---|70-657/CtMtr2
365-564/CtMtr2
93-360
% Bound
Protein Conc. (mM)

## Slide 2
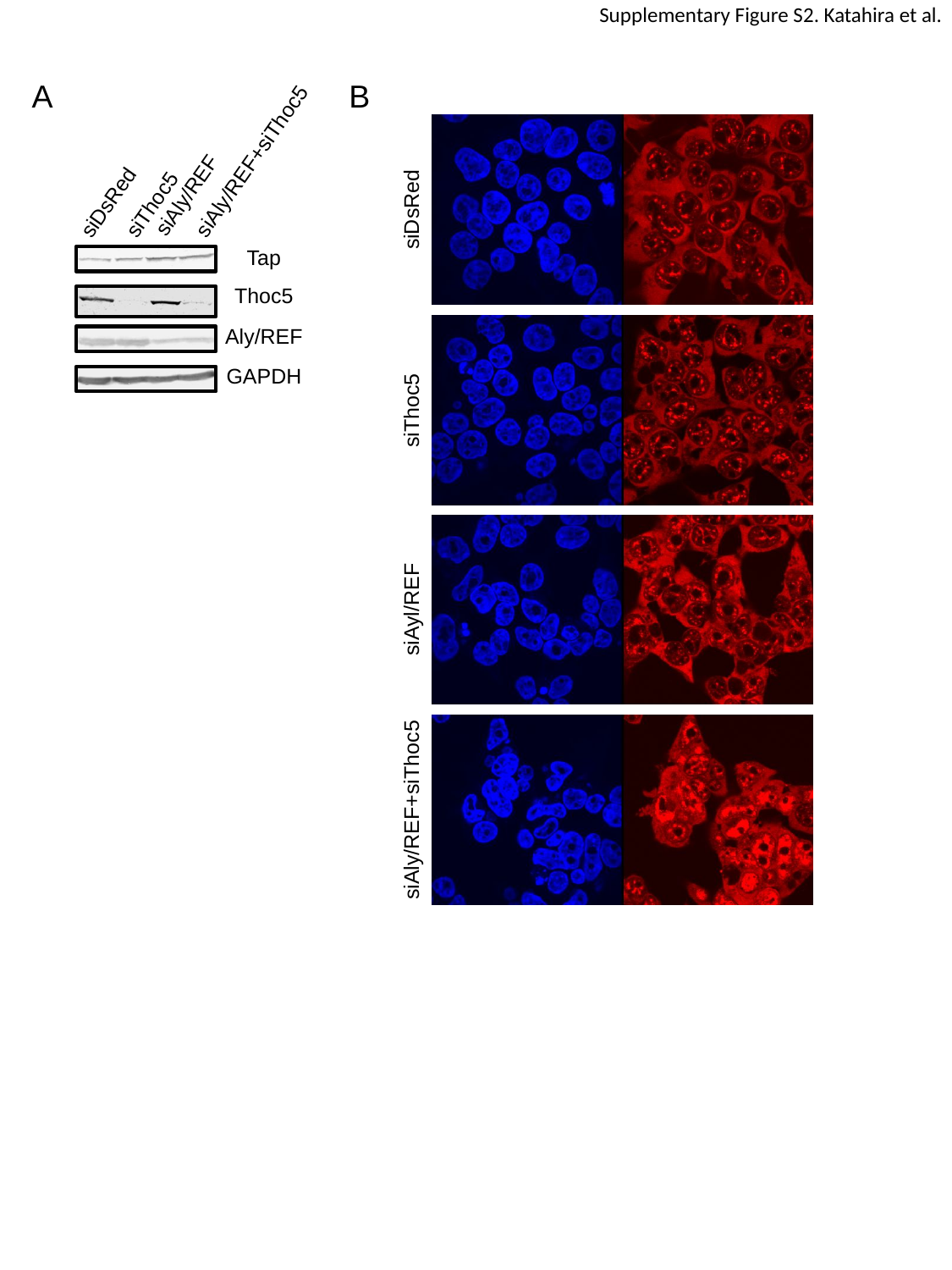

Supplementary Figure S2. Katahira et al.
siAly/REF+siThoc5
siAly/REF
siDsRed
siThoc5
Tap
Thoc5
Aly/REF
GAPDH
A
B
siDsRed
siThoc5
siAyl/REF
siAly/REF+siThoc5
